# Antidermatophytic activity of some newly synthesized arylhydrazonothiazoles conjugated with monoclonal antibody

**DOI:** 10.1038/s41598-020-77829-x

**Published:** 2020-11-30

**Authors:** Salama A. Ouf, Sobhi M. Gomha, Mohamed Eweis, Ahmed S. Ouf, Ihab A. A. Sharawy, Sulaiman A. Alharbi

**Affiliations:** 1 Botany & Microbiology Department, Faculty of Science, Cairo University, Giza, 12613 Egypt; 2 Chemistry Department, Faculty of Science, Cairo University, Giza, 12613 Egypt; 3 Department of Chemistry, Faculty of Science, Islamic University in Almadinah Almonawara, Medina, 42351 Saudi Arabia; 4 Kasr Al Ainy Medical School, Faculty of Medicine, Cairo University, Cairo, Egypt; 5 Department of Botany & Microbiology, College of Science, King Saud University, P.O Box 2455, Riyadh, 1145 Saudi Arabia

**Keywords:** Drug discovery, Microbiology, Medical research, Chemistry

## Abstract

A new series of 5-arylhydrazonothiazole derivatives **5a–d** has been synthesized, elucidated, and evaluated for their antidermatophytic activity. The minimum inhibitory concentration (MIC) and minimum fungicidal concentration (MFC) of the newly synthesized products were investigated against 18 dermatophyte fungal isolates related to *Epidermophyton floccosum*, *Microsporum canis*, and *Trichophyton rubrum*. The morphological alterations induced by the synthesized derivatives singly or conjugated with the monoclonal antibody were examined on spores of *T. rubrum* using a scanning electron microscope. The efficacy of synthesized derivative **5a** applied at its respective MFC alone or conjugated with anti-dermatophyte monoclonal antibody 0014 in skin infection treatment of guinea pigs due to inoculation with one of the examined dermatophytes, in comparison with fluconazole as standard reference drug was evaluated. In an in vivo experiment, the efficiency of **5a** derivative conjugated with the antibody induced 100% healing after 45 days in the case of *T. rubrum* and *M. canis*-infected guinea pigs.

## Introduction

Dermatophytes fungi are common pathogens that cause cutaneous mycoses, including skin, nails, hairs, and other superficial keratinized tissues in humans and animals. For several decades, the spread of these diseases in people affected by dermatophytes has rapidly increased, probably due to the increase in immune-deficient patients and the aging of several communities, particularly the people suffering from chronic diseases^1,2^. Although dermatomycosis is generally non-fatal in most cases condition; however, some causing significant severe morbidity or even mortality and is difficult to be eliminated as most of antifungal drugs used in their treatment often require long-term treatment^3^.

Focusing on dermatomycosis, there are several available fungicidal compounds currently used to control the spread of these pathogens. One of the preferred antifungal agents commonly encountered for the treatment of fungal infection are azoles and their derivatives. Azoles possess a broad range of biological activities including 2-(cyclopropylmethylidene)hydrazinyl)thiazole screened as anti-candidiasis^4^, vinyl ether-containing azole derivatives exhibiting potent anti trichophytosis against *Trichophyton rubrum* comparable with the reference antifungals as fluconazole, itraconazole, omoconazole, voriconazole, and amphotericin B^5^, 1-[[4-(4-bromophenyl)-5-(2-furyl)-4*H*-1,2,4-triazole-3-yl]mercaptoacetyl]-4-alkyl/aryl-3-thiosemicarbazides displayed different inhibitory levels against *Microsporum gypseum* NCPF580, *M. canis*, *Trichophyton mentagrophytes*, *T. rubrum*, and *Candida albicans*^6^, thiazole compounds having powerful antibacterial activity against multidrug-resistant human and animal strains of *Staphylococcus aureus*^7^, 4-aryl/chloroalkyl-2-(2,3,5-trichlorophenyl)-1,3-thiazoles showing potent antibacterial against *Pseudomonas aeruginosa* and *Escherichia coli*^8^.

Azoles are heterocyclic compounds with triazole or imidazole moiety in their configuration have been applied in human and veterinary pharmaceuticals uses as well as in agriculture to control plant pathogens. Azoles have a different mode of actions that result in fungal inhibition. Zonios and Bennett^9^ indicated that azoles can retard ergosterol synthesis in the fungal cell membrane by blocking lanosterol 14-alpha-demethylase enzyme related to the key translation of lanosterol to ergosterol. This result in increasing the membrane permeability and consequently, cell death. Moreover, the azoles may reduce the fungal metabolism by inhibiting cytochrome P450 enzyme and drug-drug interactions^10^.

As one of the methodologies for the prevention of fungal diseases is the quick treatment of mycological infection with the proper antifungal agent as soon as the etiological agent is identified. In either case, there is a need for a new antifungal agent that explicitly represses a particular fungal species and will help to overcome pathogen invasion.

The distinctive features of thiazole compounds in terms of possible adjustments in their configuration are worth studying and require the preparation of some less harmful and more effective drugs. Preliminary antifungal evaluation of a series of 14 arylazothiazole and 4 arylhydrazothiazole derivatives was tested in previous research by the authors^11^. As these derivatives are encouraging, the current study offers a summary of the synthesis and efficacy of the four derivatives of arylhydrazothiazole against certain cutaneous fungi.

## Materials and methodologies

### Investigated compounds

**Figure 1:**
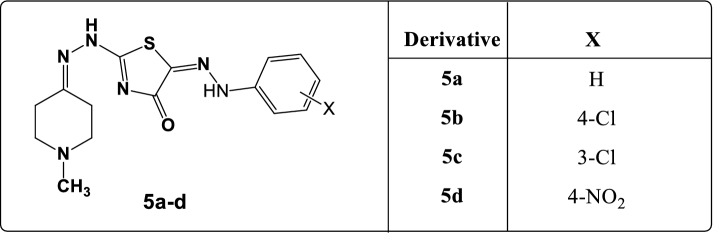
Investigated arylhydrazonothiazole derivatives **5a–d**.

The investigated arylhydrazonothiazoles **5a–d,** and their structural formulae are revealed in Fig. 1.

### Test organisms

Six different dermatophyte strains from each of *Epidermophyton floccosum*, *Microsporum canis*, and *Trichophyton rubrum* were tested in this research. The test isolates were chosen from the identified stock specimens of the first author and were isolated from patients with superficial clinical infections with different tinea infections admitted to dermatological and microbiological laboratories in Cairo, Egypt^12^. The diagnosis and identification of species were earlier established by direct microscopy, culture features, biochemical characteristics and/or a PCR assay. The identification was confirmed by Assiut University Mycological Centre, Assuit, Egypt. Detailed information of the tested strains was recorded in the Supplementary Table S1. The patients were not directly involved in this study. The inoculums were prepared from 10-day-old cultures. The isolates were maintained in Sabouraud dextrose agar (SDA) (Oxoid) with 20% glycerol, preserved at − 80 °C and cultured on potato dextrose agar (PDA) (Oxoid) and SDA plates/slopes incubated at 30 °C before investigation.

### Thiazole antibody conjugates

The antibody was manufactured and kindly delivered from Xceltis, Mannheim, Germany. The monoclonal antibody 0014 distinguishes an antigen of about 20,000 kDa MW of the fungal wall in several dermatophyte species, including those used in this research. The diluted antibody (1:100) was conjugated on the tested arylhydrazothiazole derivative with reference to the technique designated by Weissenböck et al.^13^. The conjugation was achieved by activating the carboxyl groups on the surface of the compound being examined and then binding the active carboxyl groups via the amino group of the antibody.

### Minimum inhibitory concentration (MIC) and minimum fungicidal concentration (MFC)

The MIC of the thiazole derivatives of different isolates were assessed by a broth microdilution assay according to the Clinical and Laboratory Standards Institute (CLSI) M38-A2 guidelines^14^. The inoculum suspension of each test fungal isolate was prepared by scraping the surface of the colonies previously incubated for 10 days on SDA at 30 °C. The suspension was adjusted using a double beam UV–visible spectrophotometer (ELICO, SL 159 UV–VIS spectrophotometer) until attainment of adequate transmittance and the count of the colony-forming units (CFU/mL) was confirmed microscopically by hematocytometer and by counting the emerged colonies from 10 mL of the prepared suspension on SDA. Spore suspension (100 μL) containing 10^5^/mL of each tested fungus were inoculated into 96 well microdilution plates containing 100 μL of the twofold dilution of each thiazole derivative without antibody conjugation or with conjugated anti-dermatophyte monoclonal antibody 0014. The thiazole derivatives were essentially solubilized in dimethyl sulfoxide (DMSO). Fluconazole (Sigma-Aldrich, Sintra, Portugal) as antifungal drug was used as standard following a similar manner. The microdilution plates were incubated at 30 °C, and the results were measured daily until the growth just appears in the thiazole free control. The run of each assay was done in triplicate. The concentrations required to induce 50% inhibition in fungal growth, are defined as MIC. After 72 h of incubation, a definite volume (20 μL) of the well showing complete inhibition was subcultured onto SDA plates. The plates were incubated at 35 °C until growth was apparent in the growth control subculture. The MFC was the lowest thiazole concentration that did not show any change or revealed three colonies or less to obtain approximately 99 to 99.5% killing activity.

### Scanning electron microscope (SEM)

The effect of the most efficient synthesized thiazole derivative **5a** singly or conjugate with the anti-dermatophyte monoclonal antibody 0014 on spores of *T. rubrum* as an example dermatophyte was investigated using SEM according to the method described by Ouf et al.^15^. After successive centrifugation, the spores suspended in sterile demineralized water were distributed in six sterile tubes, each containing 1 mL suspension. Arylhydrazothiazole derivative **5a** (1 µg/mL) used to treat two tubes, the other two tubes with the same concentration of the same derivative but conjugated with the antibody; In the last two tubes, the spore suspension remained without treatment and acted as a control. Approximately 25 mL aliquots of untreated and treated spore suspensions were dispersed on polystyrene sheets with a diameter of 1.0 cm and dried for 24 h at room temperature. The sheets were then mounted and gold-plated on a metallic support, then were observated by SEM at 20.0 kV with a FEI/Philips XL30 microscope.

### Animal infection model experiments

This trial was set up to examine the possibility of utilizing one of the synthesized arylhydrazothiazole derivative **5a** or fluconazole conjugated with the anti-dermatophyte monoclonal antibody 0014 in curative the dermatophyte-inoculated guinea pigs. Housing, handling and treatment of animals was done in compliance with Egyptian national legislation on the care and use of laboratory animals and approved by Cairo University, Egypt's Animal Ethics Committee (Approval No. 3006/434). The complete plan of the experimentation was defined in a previous publication by authors^11^. The infection inoculum containing 1 × 10^4^ spores/mL of the dermatophyte was smeared on dorsal side of the tested pigs and induced clear infected areas 5 days after inoculation. One millilitre of thiazole derivative **5a** without antibody conjugation or with conjugation with the antibody was applied at its MFC to each of the infected areas. Fluconazole at respective MFC was used as reference drug. The treatment was applied for 15 days at 3-day intervals for both experiments and the study was terminated after 45 days of treatment. By the end of the experiments, the recovered pigs were clinically and microbiologically examined to ensure that they are free from any site of infection or outstanding spores. The recovery was assessed as a percentage. Each experimental group contained at least five animals and the experiment was replicated one more time.

### Statistical analysis

Analysis of variance (ANOVA) and F-tests was used to determine significant differences among the treatment means at *p * ≤ 5%. Multiple comparisons were made by the least significant difference (LSD).

### Synthesis of the tested hydrazonothiazoles

**Scheme 1:**
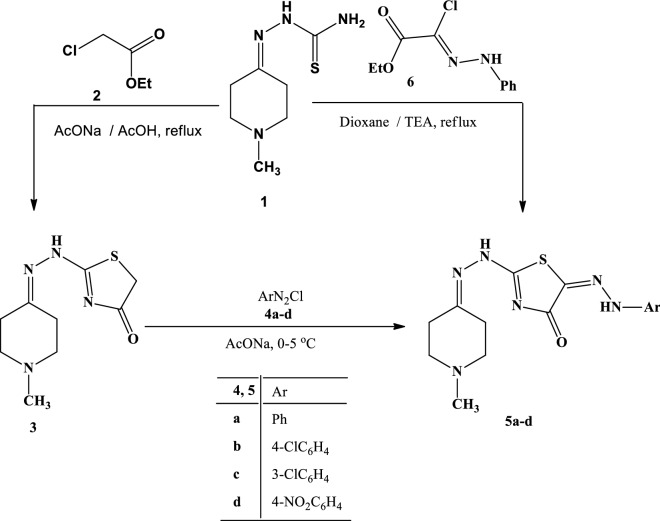
Synthesis of arylhydrazonothiazole derivatives **5a–d**.

2-(1-Methylpiperidin-4-ylidene)hydrazinecarbothioamide (**1**) was synthesized according to the reported procedure^11^ and then used as a starting material to obtain different heterocyclic compounds. Thus, refluxing of **1** with ethylchloroacetate **2** in AcOH in the presence of AcONa for 4 h, gave the new 2-(2-(1-methylpiperidin-4-ylidene)hydrazinyl)thiazol-4(5*H*)-one **(3)** (Scheme 1). Structure **2** was elucidated based on spectral data (^1^HNMR, IR, mass) and elemental. For example, ^1^H NMR spectrum for **3** showed a singlet signal at *δ* 4.23 ppm due to the methylene protons of thiazole ring and another singlet at *δ* 11.16 ppm assignable to the NH proton (D_2_O exchangeable). Its IR spectrum showed an absorption peak at 1709 cm^−1^ due to C=O stretching vibrations, and another band at υ = 3422 cm^−1^ attributed to the NH group. Mass spectrum of derivative **3** showed the presence of the molecular ion peak at m/z 226.

The targeted products, arylhydrazono-thiazolone derivatives **5a–d** were prepared from the coupling of thiazolone derivative **3** with arenediazonium chlorides **4a–d** in pyridine at 0–5 °C (Scheme 1). The products **5a–d** were satisfactorily confirmed by ^1^H-NMR, IR, MS, and their elemental analyses. The ^1^H-NMR spectra of derivatives **5a–d** assured the two NH protons as two singlet signals at *δ* ~ 9.27 and 10.79 ppm. The IR spectra revealed in each case three bands at υ = 1624, 3258 and 3423 cm^−1^, which are assigned to the carbonyl group and the 2NH groups. Also, the mass spectra of the products **5a–d** showed the molecular ion peak at the exact m/z values.

Products **5a–d** were verified by the alternative synthesis of product **5a** via refluxing compound **1** with hydrazonoyl chloride (**6**) in the presence of TEA, which gives identical products in all cases respects with the product resulting from the reaction of compound **3** with **4a** (Scheme 1).

## Experimental

### Materials and reagents

The solvents, ethanol, methanol, acetic acid, and dioxane were attained from El Nasr pharmaceutical and chemical company, Egypt. Acetyl acetone, ethyl chloroacetate, ethyl acetoacetate, and pyridine were attained from Aldrich chemical Co. Ltd. Donset, England. Sulfuryl chloride was Merck product Schuchardt, Munchen. Sodium acetate.3H_2_O, thiosemicarbazide were purchased from British Drug House Ltd. London.

### Equipment

The measurement of melting points, IR, NMR, ^1^H and mass spectra as well as elemental analyses and the employed instruments were measured according to Sayed et al.^16^ and present in the Supplementary Materials S1.

### Synthesis of compound 3

Ethyl chloroacetate (**2**) (1.22 g, l0 mmol) was refluxed for 6 h with thiosemicarbazone derivative (**1)** (1.86 g, 10 mmol) in AcOH (20 mL) having AcONa (3.3 g, 40 mmol). The obtained precipitate was filtered off, and finally recrystalized to give the thiazolone derivative **3** as yellow solid (72% yield); mp 202–204 °C (EtOH); IR (KBr): *v*/cm^−1^ 3422 (NH), 2938 (CH), 1709 (C=O), 1599 (C=N); MS *m/z* (%): 226 (M^+^, 14), 184 (19), 117 (80), 75 (79), 64 (100); ^1^H-NMR: *δ* 1.29–1.34 (2CH_2_, 4H, m), 2.90–3.01 (2CH_2_, 4H, m), 3.55 (NCH_3_, 3H, s), 4.23 (CH_2_, 2H, s), 11.16 (NH, 1H, s); ^13^C-NMR: *δ* 21.96, 24.27, 38.47 (CH_2_), 42.47 (CH_3_), 54.68 (CH_2_), 152.48, 155.59 (C=N), 177.26 (C=O). Anal.Calcd for C_9_H_14_N_4_OS (226.09): C, 47.77; H, 6.24; N, 24.76. Found C, 47.69; H, 6.20; N, 24.85%.

### General procedure for coupling of thiazolone 3 with arenediazonium chlorides 4a–d

The arenediazonium chloride solution was added slowly to a cold 10 mL pyridine containing thiazolone derivative **3** (0.226 g, 1 mmol). After completeness, the obtained precipitate was filtered and finally recrystallized from EtOH or DMF to give pure yellow crystalline products **5a–d**.

**Compound 5a:** 68% yield; mp 170–172 °C; ^1^H-NMR: *δ* 1.17 (2CH_2_, 4H, m), 3.01 (2CH_2_, 4H, m), 3.54 (NCH_3_, 3H, s), 7.14–7.94 (Ar–H, 5H, m), 9.27, 10.79 (2NH, 2H, 2br s); ^13^C-NMR: *δ* 22.70, 25.39 (CH_2_), 41.27 (CH_3_), 55.07 (CH_2_), 117.50, 123.28, 125.94, 130.04, 142.05, 151.52, 157.40 (Ar–C and C=N), 172.19 (C=O); MS *m/z* (%): 330 (M^+^, 26), 297 (28), 170 (100), 137 (52), 75 (80); IR (KBr): *v* 3423, 3258 (2NH), 3041, 2923 (CH), 1624 (C=O), 1597 (C=N) cm^−1^. Anal.Calcd for C_15_H_18_N_6_OS (330.13): C, 54.53; H, 5.49; N, 25.44. Found C, 54.38; H, 5.40; N, 25.31%.

**Compound 5b:** 68% yield; mp 190–192 °C; ^1^H-NMR: *δ* 1.19 (2CH_2_, 4H, m), 3.03 (2CH_2_, 4H, m), 3.55 (NCH_3_, 3H, s), 7.03–7.88 (Ar–H, 4H, m), 9.27, 10.63 (2NH, 2H, 2br s); MS *m/z* (%): 366 (M^+^ + 2, 6), 364 (M^+^, 17), 340 (28), 113 (42), 280 (51), 152 (44), 75 (100); IR (KBr): *v* 3428, 3252 (2NH), 2980, 2922 (CH), 1632 (C=O), 1597 (C=N) cm^−1^. Anal.Calcd for C_15_H_17_ClN_6_OS (364.09): C, 49.38; H, 4.70; N, 23.03. Found C, 49.30; H, 4.63; N, 22.85%.

**Compound 5c:** 65% yield; mp 171–173 °C; ^1^H-NMR: *δ* 1.18 (2CH_2_, 4H, m), 3.07 (2CH_2_, 4H, m), 3.56 (NCH_3_, 3H, s), 7.28–7.58 (Ar–H, 4H, m), 9.12, 10.96 (2NH, 2H, 2br s); MS *m/z* (%): 366 (M^+^ + 2, 2), 364 (M^+^, 7), 354 (28), 297 (100), 182 (53), 125 (69), 74 (95); IR (KBr): *v* 3426, 3373 (2NH), 2974, 2923 (CH), 1638 (C=O), 1594 (C=N) cm^−1^. Anal.Calcd for C_15_H_17_ClN_6_OS (364.09): C, 49.38; H, 4.70; N, 23.03. Found C, 49.29; H, 4.86; N, 22.90%.

**Compound 5d:** 67% yield; mp 195–197 °C; ^1^H-NMR: *δ* 1.18 (2CH_2_, 4H, m), 3.07 (2CH_2_, 4H, m), 3.56 (NCH_3_, 3H, s), 7.39–8.28 (Ar–H, 4H, m), 9.86, 11.23 (2NH, 2H, 2br s); MS *m/z* (%): 375 (M^+^, 28, 271 (100), 167 (50), 103 (98), 77(82); IR (KBr): *v* 3426, 3373 (2NH), 2974, 2925 (CH), 1638 (C=O), 1596 (C=N) cm^−1^. Anal.Calcd for C_15_H_17_N_7_O_3_S (375.11): C, 47.99; H, 4.56; N, 26.12. Found C, 47.75; H, 4.46; N, 26.03%.

### Alternate synthesis of 5a

Hydrazonoyl chloride (**7**) (0.226 g, 1 mmol) was refluxed with thiosemicarbazone derivative **1** (0.186 g, 1 mmol) in dioxane (30 mL) containing 0.1 g triethylamine for 8 h. The formed product was recrystallized from DMF to give **5a**.

### Ethical approval and consent to participate

The housing of the animal, handling situations, and care was achieved according to the Egyptian national legislation for the care and use of laboratory animals and approved by Cairo University, Egypt animal ethics committee (Approval No. 3006/434). All procedures performed in studies involving animals were carried out in accordance with the ethical standards of the institutional and/or national research committee and with the 1964 Helsinki declaration and its later amendments or comparable ethical standards. The patients were not directly involved in the study.

## Results and discussion

### MIC and MFC

The MIC of the synthesized arylhydrazothiazole derivatives for 18 dermatophyte isolates related to three species revealed that derivative **5a** is the most inhibitory compared to the other derivatives or to the fluconazole as the reference drug (Table 1). The MIC of derivative 5a recorded 4–8, 0.5–1, and 1 µg/mL for *E. floccosum*, *M. canis* and *T. rubrum*, respectively, compared to 16–32, 16 and 32 µg/mL for fluconazole for the same species, respectively (Table 1). The other derivatives (may be **6b**, **6c**, **6d**) were found to be less effective towards the test isolates compared to fluconazole. The incorporation of the unsubstituted phenyl ring in dioxane was found to induce a more inhibitory effect than chloro- or nitro-substituted phenyl ring. Compounds containing dioxane ring exhibited different antimicrobial activities^11,17,18^. Ouf et al.^11^ tested fourteen synthesized thiazole derivatives for their potential activity against some cutaneous fungi*.* They found that the efficacy of the compound mostly depends on the side chains of the core compound. The data showed that the efficiency of the synthesized derivatives was analogous or equivalent with fluconazole as a reference drug**.** The conjugation of the synthesized thiazole derivative with the antibody enhanced the inhibitory efficacies of the thiazole derivatives. Consequently, it was effective in lowering MIC recording 0.5–1, 0.25, and 0.5 µg/mL in the case of derivative **5a** for *E. floccosum*, *M. canis* and *T. rubrum*, respectively. The conjugation of the synthesized derivatives with specific anti-dermatophyte monoclonal antibody enables and directs the synthesized compounds to recognize and detect the hypha or spore cell in the dermatological sample to differentially bind to the fungal cell wall polysaccharide antigenic receptor of the target dermatophyte^19,20^. Nejadmoghaddam et al.^21^ indicated that the design of antibody–drug conjugates is an efficient targeting agent for tumor cell in clinical applications. This approach acts as a targeting agent and a nanoscale carrier to deliver a therapeutic dose of cytotoxic cargo into desired unhealthy cells.

**Table 1:** Range of minimum inhibitory and minimum fungicidal concentrations (MIC, MFC, respectively) of synthesized arylhydrazothiazole derivatives conjugated with anti-dermatophyte monoclonal antibody 0014 against different dermatophyte fungal isolates measured as µg/mL.

Arylhydrazo-thiazole derivative	Dermatophytes (6 isolates tested for each species)											
	*Epidermophyton floccosum*				*Microsporum canis*				*Trichophyton rubrum*			
	No antigen		Conjugated with antigen		No antigen		Conjugated with antigen		No antigen		Conjugated with antigen	
	MIC	MFC	MIC	MFC	MIC	MFC	MIC	MFC	MIC	MFC	MIC	MFC
**5a**	4–8	16–32	0.5–1	1–4	0.5–1	2–4	0.25	1	1.0	2.0	0.5	0.5–1
**5b**	128–256	> 512	16–32	32–64	16	32	8	16	32	64	8	8–16
**5c**	128–256	> 512	16	64	16–32	64	8	16–32	16	64	8	16–32
**5d**	128	512	16–32	32–64	128	256	16	32	128	> 256	64	128
Fluconazole	16–32	64	4–8	8–16	16	32–64	4–8	16	32	64	16	8–16

The values of MFC showed a general drop with the treatment of the antibody conjugated thiazole derivatives in the case of all tested fungal isolates reaching 1–4, 1, and 1.0 µg/mL compared to 16–32, 2, 2–4 µg/mL for the non-conjugated derivative in the case of *E. floccosum*, *M. canis* and *T. rubrum*, respectively for **5a** derivative. This is probably may be due to the definite focusing, rapid delivery, and the more contact of the synthesized derivative and the receptor point (s) in the cell wall of the fungal isolate. The developing literature proposes that the antibody internalization and transport of the toxic drug to the target cells aids as the key mechanism of action for antibody^22^.

### Scanning electron microscopy (SEM)

**Figure 2:**
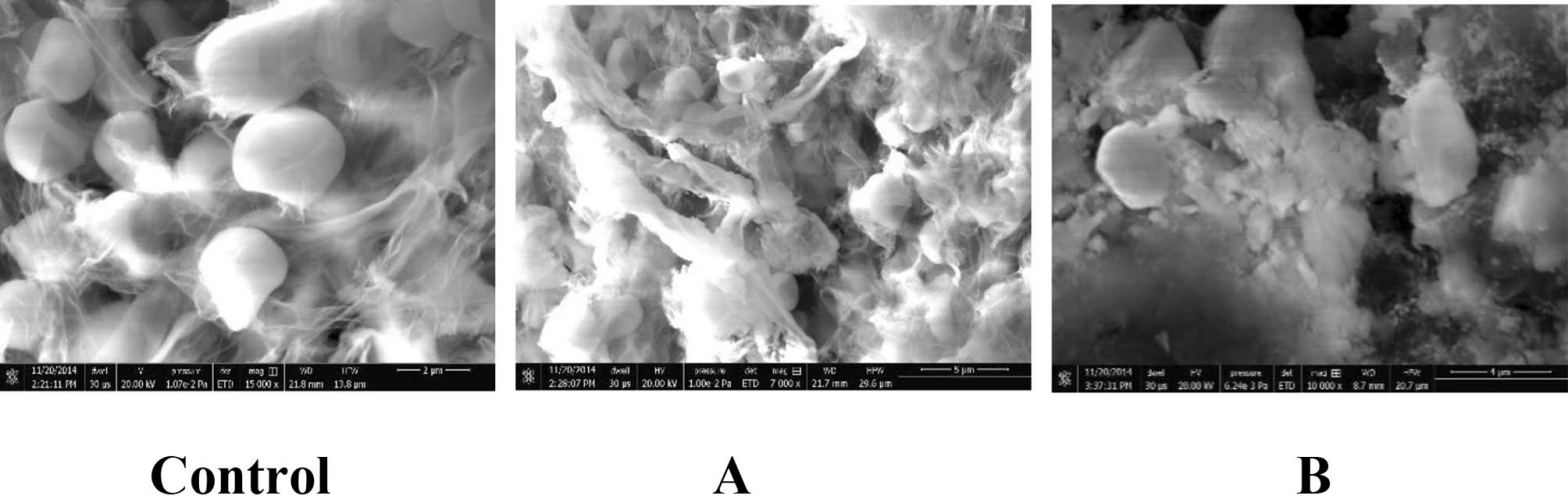
SEM images of *T. rubrum* treated with 1 µg/ ml of arylhydrazothiazole derivative 5a singly (**A**) or conjugated with anti-dermatophyte monoclonal antibody 0014 (**B**) as compared to control.

SEM examination was used for the evaluation of morphological changes that occurred in *T. rubrum* as an example. The SEM images revealed variation in the structural pattern of the mycelial and spore structure of 1 µg/mL arylhydrazothiazole derivative **5a**-treated specimen (Fig. 2). The treated spores appeared irregular, with evident destruction and losing their smoothness. The hyphae appear contracted, empty of the cytoplasmic content, wrinkled, missing its turgidity, and with disrupted walls. The upshot of the **5a** derivative is more pronounced and evident in the case of antigen conjugation. Romagnoli et al.^23^ indicated that 5-amino-3-methyl-1-phenylpyrazole-4-tiocynate induced relevant common alterations and distorted or swollen apexes to *T. rubrum* at 50 µg/mL. Pawar et al.^24^ demonstrated significant morphological alterations and cell wall defects were seen in indole–triazole–amino acid treated *C. albicans* cells with the combination of ketoconazole.

### Animal infection model experiments

**Figure 3:**
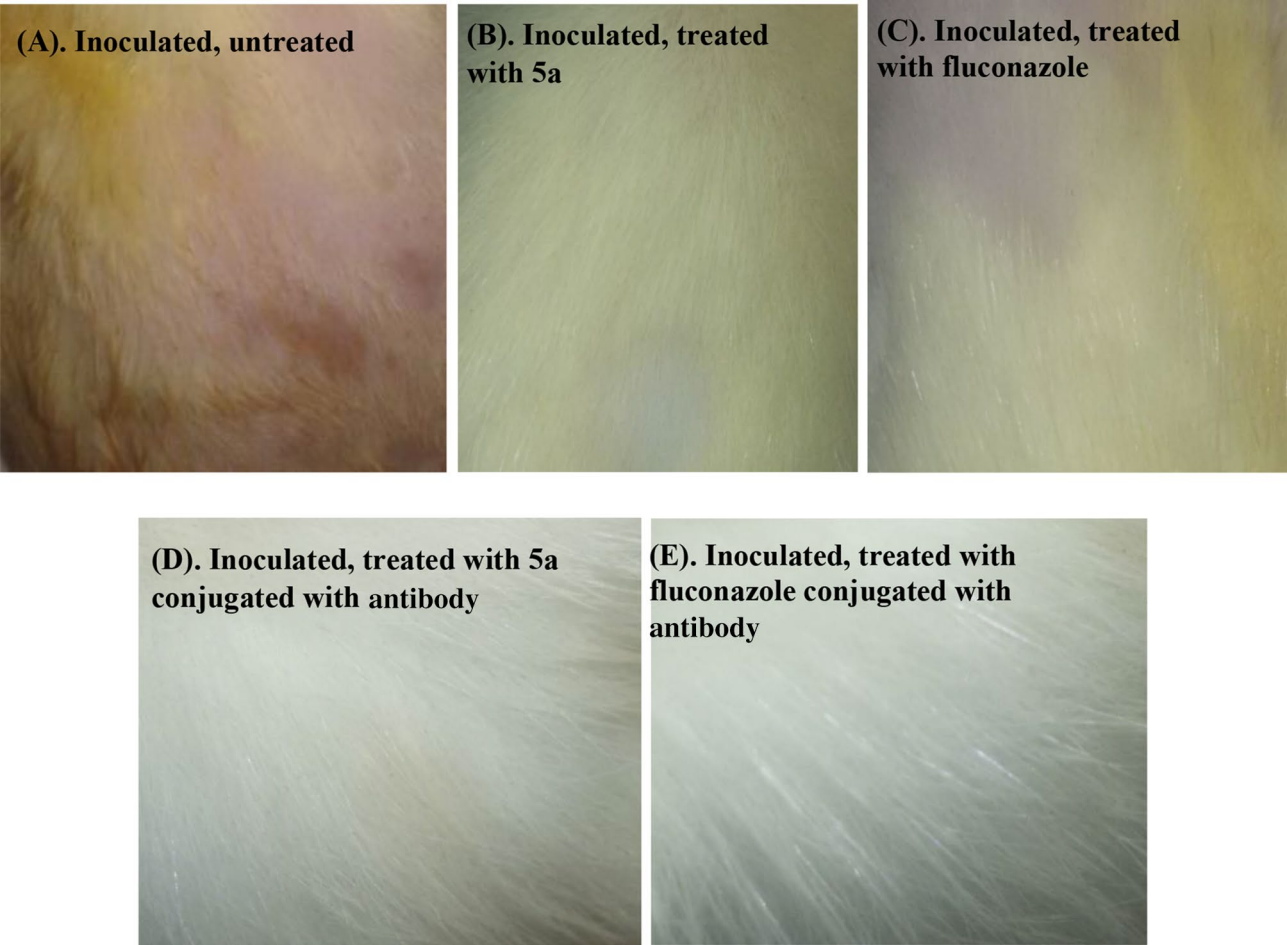
Photographs of guinea pig skin showing the efficacy of synthesized arylhydrazothiazole derivative (**5a**) alone applied at the respective MFC (**B**) and conjugated with anti-dermatophyte monoclonal antibody 0014 (**C**) in treatment of skin infection of guinea pigs due to inoculation with *Trichophyton rubrum*, 45 days post inoculation as compared with fluconazole. Treatments (**B**) and (**C**) showed residual infection symptoms, while treatments (**D**) and (**E**) revealed complete recovery. The application of monoclonal antibody alone did not show any effect.

The efficacy of synthesized arylhydrazothiazole derivative **5a** applied at its respective MFC (1 µg/mL) alone or conjugated with anti-dermatophyte monoclonal antibody 0014 in treating guinea pigs infected with the investigated dermatophytes, as compared with fluconazole as standard fungicide is shown in Table 2 and Fig. 3. The thiazole derivative **5a** was efficient in healing the guinea pigs previously inoculated with *E. floccosum*, *M. canis* or *T. rubrum*. The rate of recovery varied according to the resistance and susceptibility of the dermatophyte fungus to 5a derivative. *M. canis* was the most sensitive to the tested derivative, followed by *T. rubrum* and *E. floccosum*. The percentage healing recorded 77.6, 90.5, and 84.6 in the case of *E. floccosum, M. canis* and *T. rubrum*, respectively. The percentage values are mostly compatible to those obtained with the reference drug. The efficiency of **5a** derivative significantly increased with all investigated fungi compared with fluconazole reaching 100% curing on applying **5a** derivative conjugated with the monoclonal antibody 0014 in the case of *M. canis*-infected guinea pigs. Ouf et al.^25^ indicated that the silver nanoparticles are more efficacious and induced high % curing of *M. canis*–infected guinea pigs when the nanoparticles are conjugated with the antibody. The authors suggest that the antibody helps in recognizing dermatophyte or the pathogen components and directs the tested thiazole to the site of infection. The possible technique of in vivo application against dermatophytosis was achieved by the use of synthetic thiazole conjugated with the antibody. The computational information and prediction of the bioactivity concerning the arylhydrazothiazole **5a** previously indicated its authentication as a pharmacologic drug being it does not induce significant violation and verifies fitting correlated to absorption, distribution, excretion, and metabolism^11^. The results recommend that compound **5a** might be a promising candidate and necessitates further potential in vivo investigations for its use in topical medication against Tinea infections.

**Table 2:** Efficacy of synthesized arylhydrazothiazole derivative 5a applied at its respective MFC alone or conjugated with anti-dermatophyte monoclonal antibody 0014 in treatment of skin infection of guinea pigs due to inoculation with a dermatophyte, 45 days post inoculation as compared with fluconazole as standard fungicide applied at MFC respective dose for each fungus.

Treatment	Percent healing					
	Compound **5a**			Fluconazole		
	*E. floccosum*	*M. canis*	*T. rubrum*	*E. floccosum*	*M. canis*	*T. rubrum*
Inoculated, untreated	0.0	0.0	0.0	0.0	0.0	0.0
Inoculated, treated with thiazole compound	77.6 ± 3.9	90.5 ± 6.4	84.6 ± 4	72.0 ± 6.2	86.0 ± 5.0	84.8 ± 3.8
Inoculated, treated with thiazole compound conjugated with antigen	98.1 ± 4.4	100	100	82.3 ± 4.6	95.0 ± 4.4	92.3 ± 5.3

## Conclusion

The efficiency of an azole derivative as a potential agent against a dermatophyte fungus depends on its strength and how the derivative targets and recognizes the specific host. The use of a particular antibody as a carrier is an important step in focusing the toxic compound on the dermatophyte and avoiding any undesirable side effects due to the interaction with the adjacent uninfected tissues. The conjugation of the antifungal agent to the antibody is vital as a prospect in topical therapy and will add advantage because it will minimize the risk of systemic side effects. The information provided in this manuscript can be imperative in pharmacological application, at least in topical medication.

## Supplementary information

Supplementary Material.

Supplementary Tables.

## Data Availability

The datasets generated during and/or analyzed during the current study are available from the corresponding author on reasonable request.
