# Supplementary material for: Antidermatophytic activity of some newly synthesized arylhydrazonothiazoles conjugated with monoclonal antibody
Source: Sci Rep. 2020 Nov 30;10:20863. doi: 10.1038/s41598-020-77829-x (PMC7704675; doi:10.1038/s41598-020-77829-x)
Supplement: Supplementary file 1 — Supplementary Material. [file 41598_2020_77829_MOESM1_ESM.docx]

**Supplementary material (S1)**

The melting points were measured on an Electrothermal IA 9000 series digital melting point apparatus (Bibby Sci. Lim. Stone, Staffordshire, UK). IR spectra were recorded in potassium bromide discs on PyeUnicam SP 3300 and Shimadzu FTIR 8101 PC infrared spectrophotometers (Shimadzu, Tokyo, Japan). NMR spectra were measured on a Varian Mercury VX-300 NMR spectrometer (Varian, Inc., Karlsruhe, Germany). ^1^H spectra were recorded at 300 MHz and ^13^C spectra were recorded at 75.46 MHz in deuterated dimethyl sulfoxide (DMSO-*d*6). Mass spectra were run on a Shimadzu GCMS-QP1000 EX mass spectrometer (Tokyo, Japan) at 70 eV. Elemental analyses were measured using Elementarvario LIII CHNS analyzer (Germany).
