# Supplementary material for: Antidermatophytic activity of some newly synthesized arylhydrazonothiazoles conjugated with monoclonal antibody
Source: Sci Rep. 2020 Nov 30;10:20863. doi: 10.1038/s41598-020-77829-x (PMC7704675; doi:10.1038/s41598-020-77829-x)
Supplement: Supplementary file 2 — Supplementary Tables. [file 41598_2020_77829_MOESM2_ESM.docx]

Supplementary Table S1. Strains details of the clinical isolates of the tested dermatophytes selected from 30 identified stocks for each species based on their resistance to antifungal reference drugs.

| Species | Isolate number | Clinical site | Clinical type |
| --- | --- | --- | --- |
| *E. floccosum* | EF101 | groin | Tinea cruris |
|  | EF102 | Foot | Tinea pedis |
|  | EF108 | glabrous skin of arms | [tinea corporis](https://www.creative-biolabs.com/drug-discovery/therapeutics/tinea-corporis.htm) |
|  | EF122 | fingernails | Tinea unguium |
|  | Ef124 | toenails | Tinea unguium |
|  | EF129 | Legs | [tinea corporis](https://www.creative-biolabs.com/drug-discovery/therapeutics/tinea-corporis.htm) |
| *M. canis* | MC201 | Scalp | Tinea capitis |
|  | MC212 | Face | Tinea faciei |
|  | MC213 | Palms of hands | Tinea manum |
|  | MC215 | Skin | Tinea corporis |
|  | MC222 | Scalp | Tinea capitis |
|  | MC230 | Scalp | Tinea capitis |
| *T. rubrum* | TR303 | Foot | tinea pedis |
|  | TR305 | trunk | Tinea corporis |
|  | TR309 | Finger skin | Tinea manuum |
|  | TR312 | Toenail | Tinea pedis |
|  | TR315 | Foot skin | tinea pedis |
|  | TR326 | Groin | Tinea cruris |
|  |  |  |  |
